# Supplementary material for: An optimized electroporation approach for efficient CRISPR/Cas9 genome editing in murine zygotes
Source: PLoS One. 2018 May 3;13(5):e0196891. doi: 10.1371/journal.pone.0196891 (PMC5933690; doi:10.1371/journal.pone.0196891)
Supplement: S1 Fig — Sequencing results of the indicated blastocysts from Fig 1A. The expected DNA sequence of either the endogenous WT locus or the desired mutation (new BsaI restriction site) is depicted at the top. (PDF) [file pone.0196891.s001.pdf]

S1 Figure

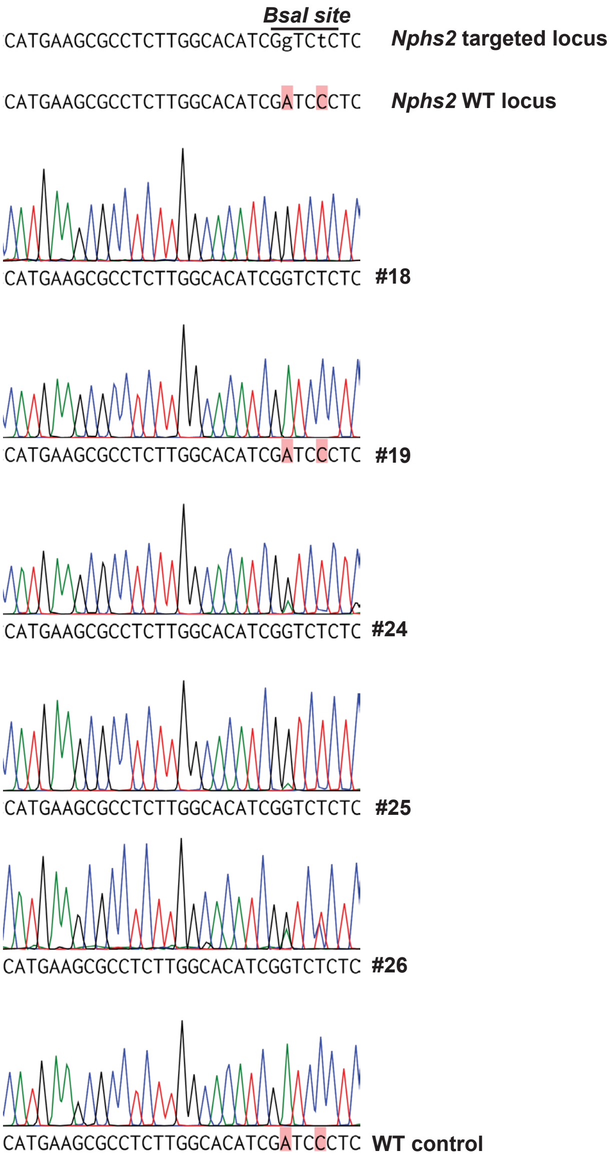
BsaI site
CATGAAGCGCCTCTTGGCACATCGGgTCtCTC
Nphs2 targeted locus
CATGAAGCGCCTCTTGGCACATCGATCCCTC
Nphs2 WT locus
CATGAAGCGCCTCTTGGCACATCGGTCTCTC
#18
CATGAAGCGCCTCTTGGCACATCGATCCCTC
#19
CATGAAGCGCCTCTTGGCACATCGGTCTCTC
#24
CATGAAGCGCCTCTTGGCACATCGGTCTCTC
#25
CATGAAGCGCCTCTTGGCACATCGGTCTCTC
#26
CATGAAGCGCCTCTTGGCACATCGATCCCTC
WT control
