# Supplementary figures and images for: An optimized electroporation approach for efficient CRISPR/Cas9 genome editing in murine zygotes
Source: PLoS One. 2018 May 3;13(5):e0196891. doi: 10.1371/journal.pone.0196891 (PMC5933690; doi:10.1371/journal.pone.0196891)

S2 Figure

A

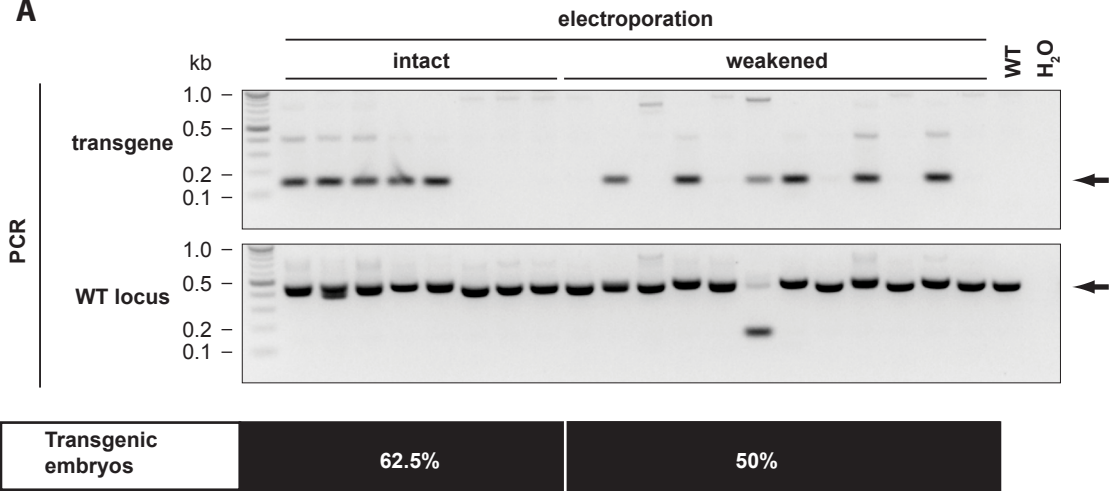

B

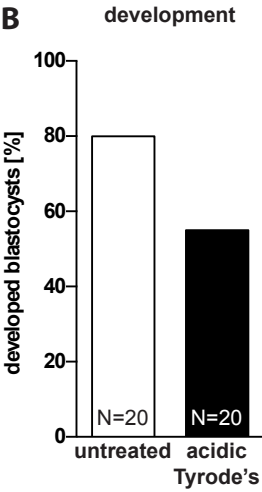

C

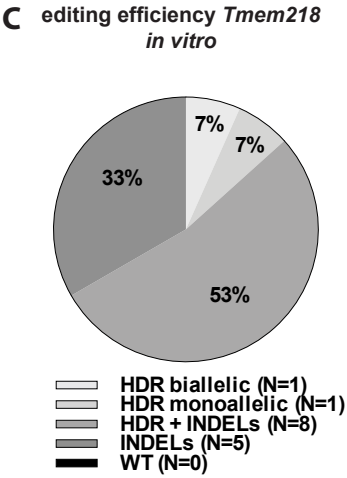

D

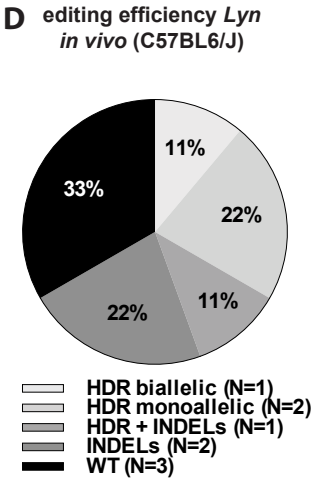

Supplement: S2 Fig — (A) Myc tag insertion into the Atp1a1 gene by electroporation. PCR genotyping and the percentage of transgenic 3.5 dpc embryos of zygotes pre-treated for 10 s with acidic Tyrode’s solution (weakened) or untreated (intact) is depicted. Arrows indicate the expected size of the band for the PCR with primers flanking the endogenous locus (WT locus) or amplifying the myc tag sequence (transgene). PCR controls from untreated embryos (WT) and without DNA template (H2O) are included. (B) Developed blastocysts upon treatment with acidic Tyrode’s solution for 10 s. (C) Quantification of Sanger sequencing of Tmem218 targeted blastocysts generated by EEZy. (D) Quantification of Sanger sequencing of biopsies from Lyn transgenic mice generated by EEZy using C57BL/6J zygotes. HDR = solely the desired mutation, HDR + INDELs = mixture of the desired mutation and INDELs, INDELs = mono- and biallelic INDELs, WT = no genome editing. N = Number of embryos/animals. (PDF) [file pone.0196891.s002.pdf]

S3 Figure

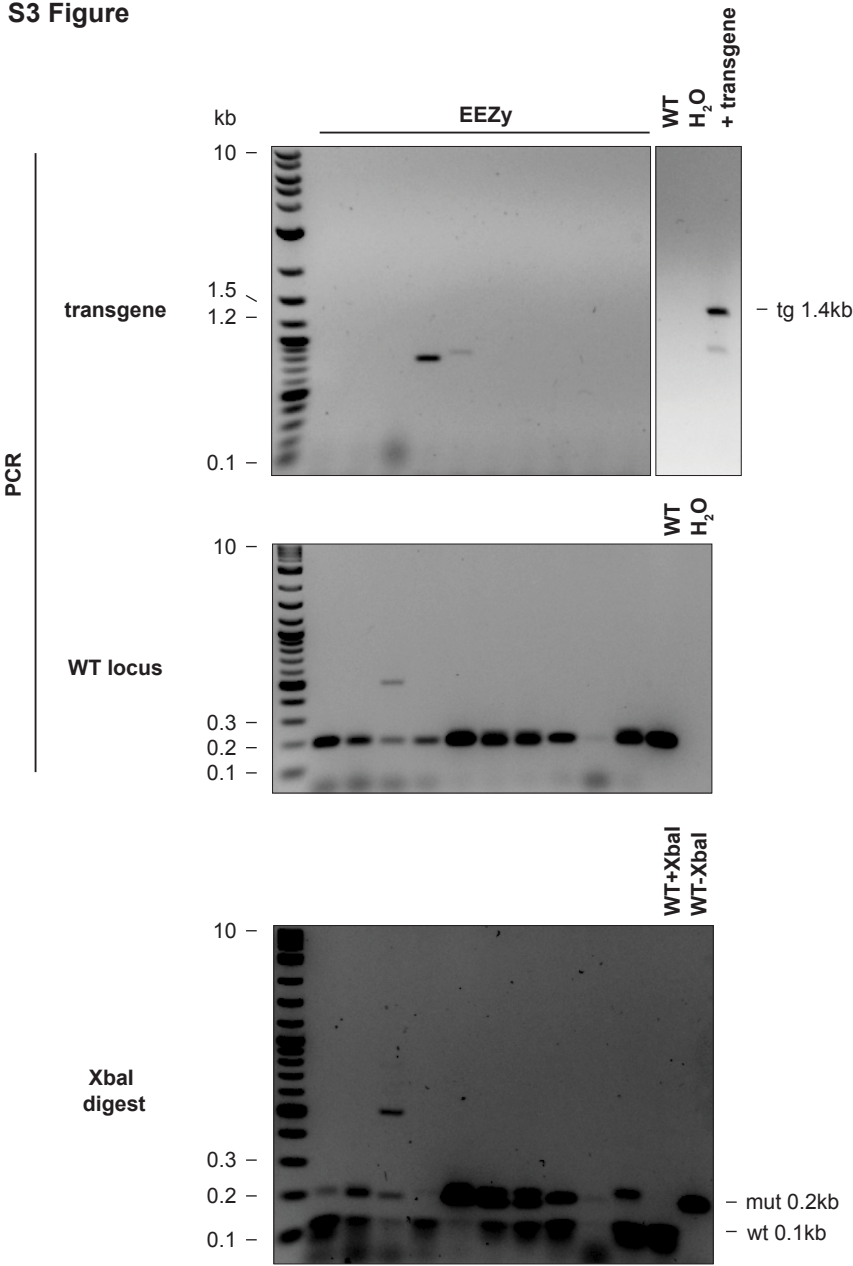

Supplement: S3 Fig — A circular Gt(ROSA)26Sor targeting vector (6 kB) harboring a Venus reporter transgene fused to a splice acceptor site (Gt(ROSA)26Sor SA-Venus) was electroporated into zygotes together with a Cas9 RNP targeting the Gt(ROSA)26Sor locus. PCR genotyping for integration of the transgene and amplification of the WT locus are depicted. PCR controls from untreated embryos (WT), without DNA template (H2O) and Gt(ROSA)26Sor SA-Venus targeted blastocysts (+transgene) are included and the expected size for amplification of the transgene (tg) depicted. Evaluation of NHEJ at the endogenous Gt(ROSA)26Sor locus is shown by partial resistance to XbaI digest (mut). Controls from PCR amplicons of WT blastocysts with (WT+XbaI) and without XbaI (WT-XbaI) are included. (PDF) [file pone.0196891.s003.pdf]

S4 Figure

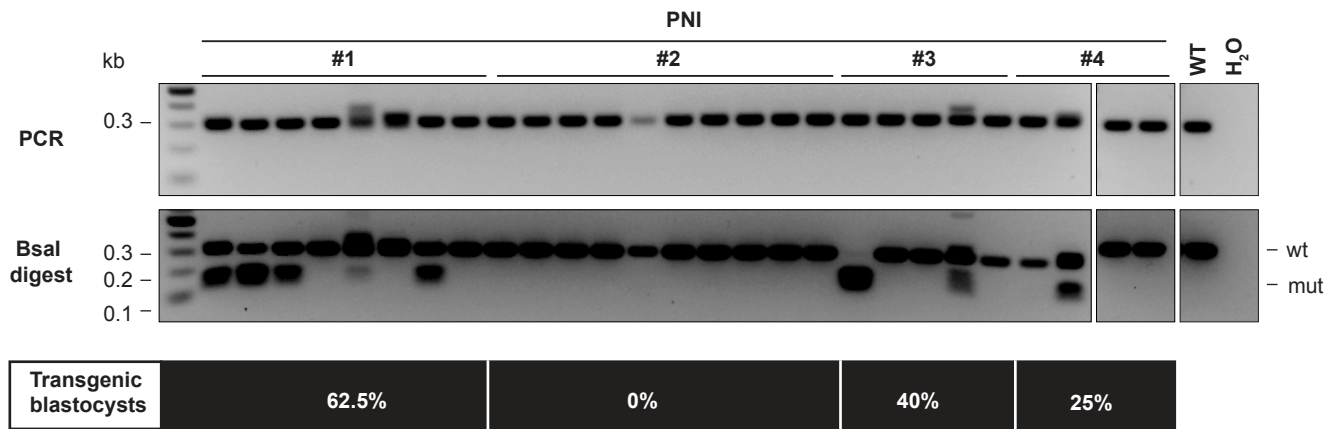

Supplement: S4 Fig — RFLP analysis of Nphs2-targeted blastocysts upon PNI. Results of four independent experiments (#1–4) with the percentage of transgenic blastocysts are depicted (n = 4). PCR controls from untreated blastocysts (WT) and without DNA template (H2O) and RFLP controls from untreated blastocysts (WT) are depicted. The data are quantified in Fig 3E. (PDF) [file pone.0196891.s004.pdf]
