# Supplementary material for: An optimized electroporation approach for efficient CRISPR/Cas9 genome editing in murine zygotes
Source: PLoS One. 2018 May 3;13(5):e0196891. doi: 10.1371/journal.pone.0196891 (PMC5933690; doi:10.1371/journal.pone.0196891)
Supplement: S1 Table — (PDF) [file pone.0196891.s005.pdf]

S1 Table. Sequences of oligonucleotides used in this study

S1A Table. Sequences of guide RNA and DNA repair templates

Target	Guide RNA sequence (5'-3')	DNA repair template sequence (5'-3')
Nphs2	UUUCCAGGAGAAUUUCAGUG	CTTCTTCTAAGCAGTCTAGCCCATGTGTCCAAAGCCATCCAGT TCCTGGTGCAAACCACCATGAAGCGCCTCTTGGCACATCGGT CTCTCATTGAAATTCTCCTGGAAAGGAAGAGCATTGCCCAAG A
Atp1a1	CAUCCGAAGUGCUACAGAAG	TCTGGGAGGCAACAGCGCTACCGTAACTACACAACTCACATC ATCGTTTGGCGGTTCCTCCAGATCCTCTTCTGAGATGAGTTTT TGTTCTGTAGCACTTCGGATGCCATAAGCCAGGAAACAAAGA ATGGCCCCGATCCACAGTAA
Lyn	CAGACAUGGUGCCGGGCUUG	TTTAGGAGGTGCAGTGCTTGAGTTCTCTGCTTTCTTTTGTCTG CCCAGGTTACTATAACAACAGCACAAAGGTGGCTGTGAGGAC ACTTAAGCCCGGCACCATGTCTGTGCAGGCATTCCTGGAAGA
Gt(ROSA) 26Sor	ACUCCAGUCUUUCUAGAAGA	SA-Venus targeting vector

S1B Table. PCR and sequencing primer and thermocycling conditions

	Primer sequence (5'-3')	Conditions	PCR rounds
Atp1a1 [nested]	fwd: GGTCTTAGGGCATTACGGGCT<br>rev: GACGACGACATCCTCCGCAT	98°C 3min; 34 cycles of [95°C-20s; 59°C-20s; 72°C-30s]; 72°C-3 min	1st
Atp1a1 [WT]	fwd: CCTCCCACTACTCCCGAAT<br>rev: TCTCCTAGGTGAGTCGATTTCT	98°C 3min; 30 cycles of [95°C-20s; 55°C-20s; 72°C-20s]; 72°C-3 min	2nd
Atp1a1 [myc]	fwd: CCTCCCACTACTCCCGAAT<br>rev: CCTCCAGATCCTCTTCTGAGAT	98°C 3min; 30 cycles of [95°C-20s; 55°C-20s; 72°C-20s]; 72°C-3 min	2nd
Nphs2	fwd: AGACGCTGTCTGCTACTACCGC<br>rev: TCCCCAGGCCAATGATGTCAC	98°C 3min; 34 cycles of [95°C-20s; 67.5°C-20s; 72°C-20s]; 72°C-3 min	2
Gt(ROSA) 26Sor [nested]	fwd: CTCGGCTAGGTAGGGGATCG<br>rev: GCATTCCAAAAGGAACCACCTT	98°C 3min; 38 cycles of [95°C-20s; 61.5°C-20s; 72°C-90s]; 72°C-3 min	1st
Gt(ROSA) 26Sor [WT]	fwd: GCCTCCTGGCTTCTGAGGACCG<br>rev: TCTGTGGGAAGTCTTGTCCCTCC	98°C 3min; 38 cycles of [95°C-20s; 66.5°C-20s; 72°C-20s]; 72°C-3 min	2nd
Gt(ROSA) 26Sor [transgene]	fwd: GGCCTCTCGAGCCTCTAGAACTATAGTG<br>rev: CAAGCTCACAAGACCTTAGGTCAGGA	98°C 3min; 38 cycles of [95°C-20s; 62°C-20s; 72°C-45s]; 72°C-3 min	1
Lyn	fwd: GCAGTGGACAATCTCCCAAGAC<br>rev: TCTCAGCACACTCTAATCCGA	98°C 3min; 34 cycles of [95°C-20s; 64.5°C-20s; 72°C-45s]; 72°C-3 min	1
Nphs2	fwd: AGACGCTGTCTGCTACTACCGC	Sanger sequencing	N.A.
Lyn	fwd: CTCCTTTAGGGGTACAGACT	Sanger sequencing	N.A.
