# Supplementary material for: An optimized electroporation approach for efficient CRISPR/Cas9 genome editing in murine zygotes
Source: PLoS One. 2018 May 3;13(5):e0196891. doi: 10.1371/journal.pone.0196891 (PMC5933690; doi:10.1371/journal.pone.0196891)
Supplement: S2 Table — (PDF) [file pone.0196891.s006.pdf]

*Fig 1B*

| no additives | | additives | |
|---|---|---|---|
| transgenic | total | transgenic | total |
| 5 | 12 | 7 | 12 |
| 11 | 17 | 8 | 17 |
| 6 | 13 | 3 | 9 |

*Fig 1C*

| no additives | | additives | |
|---|---|---|---|
| blastocysts | total | blastocysts | total |
| 13 | 25 | 12 | 23 |
| 17 | 22 | 17 | 25 |
| 13 | 15 | 9 | 14 |

*Fig 1D*

| natural mating | | | | IVF | |
|---|---|---|---|---|---|
| pgRNA | | sgRNA | | pgRNA | |
| transgenic | total | transgenic | total | transgenic | total |
| 6 | 9 | 1 | 3 | 5 | 8 |
| 3 | 6 | 2 | 3 | 8 | 9 |
| 10 | 13 | 2 | 11 | 8 | 11 |

*Fig 1E*

| natural mating | | | | IVF | |
|---|---|---|---|---|---|
| pgRNA | | sgRNA | | pgRNA | |
| blastocysts | total | blastocysts | total | blastocysts | total |
| 9 | 11 | 3 | 11 | 8 | 16 |
| 6 | 10 | 3 | 9 | 9 | 15 |
| 13 | 15 | 11 | 24 | 11 | 17 |

*Fig 1F*

| sgRNA | | pgRNA | |
|---|---|---|---|
| total | blastocysts | total | blastocysts |
| 39 | 15 | 17 | 8 |
| 34 | 9 | 17 | 10 |
| 23 | 12 | 16 | 5 |
| 27 | 8 | 16 | 5 |

*S2B Fig*

| | total | blastocysts |
|---|---|---|
| untreated | 20 | 16 |
| acidic Tyrode's | 20 | 11 |

*Fig 2A*

| + pronuclei | - pronuclei |
|---|---|
| 109 | 65 |
| 143 | 31 |
| 128 | 31 |
| 122 | 49 |
| 177 | 85 |
| 220 | 50 |
| 135 | 48 |
| 120 | 63 |
| 147 | 144 |
| 121 | 22 |
| 116 | 42 |
| 233 | 49 |
| 94 | 51 |
| 200 | 76 |
| 107 | 34 |
| 182 | 142 |
| 187 | 64 |
| 92 | 9 |
| 108 | 62 |
| 160 | 28 |
| 82 | 50 |
| 170 | 92 |
| 181 | 108 |
| 147 | 103 |
| 142 | 29 |
| 95 | 71 |
| 91 | 50 |
| 224 | 161 |
| 151 | 79 |
| 164 | 77 |
| 177 | 147 |
| 155 | 131 |
| 134 | 49 |
| 93 | 118 |
| 117 | 146 |
| 132 | 205 |
| 152 | 135 |
| 77 | 34 |
| 35 | 7 |
| 153 | 25 |
| 128 | 24 |
| 92 | 64 |
| 167 | 106 |
| 110 | 39 |
| 51 | 51 |
| 52 | 1 |
| 195 | 67 |

*Fig 2C*

| - pronuclei | |
|---|---|
| total | transgenic |
| 4 | 3 |
| 6 | 4 |
| 8 | 5 |

*Fig 2D*

| - pronuclei | |
|---|---|
| total | blastocysts |
| 36 | 4 |
| 39 | 6 |
| 30 | 8 |

***Fig 3A***

| | blastocysts | total | blastocysts | total | blastocysts | total |
|---|---|---|---|---|---|---|
| **-** | 32 | 40 | 16 | 20 | 39 | 39 |
| **Mock** | 22 | 27 | 20 | 28 | 14 | 20 |
| **CRISPR** | 27 | 40 | 21 | 26 | 12 | 20 |

***Fig 3B-D***

| PNI | | | EEZy | | |
|---|---|---|---|---|---|
| **initial** | **viable** | **blastocysts** | **initial** | **viable** | **blastocysts** |
| 20 | 17 | 8 | 20 | 20 | 11 |
| 20 | 17 | 10 | 20 | 19 | 13 |
| 20 | 16 | 5 | 20 | 18 | 11 |
| 21 | 16 | 5 | 20 | 19 | 13 |

***Fig 3D***

| PNI | |
|---|---|
| **total** | **transgenic** |
| 8 | 5 |
| 10 | 0 |
| 5 | 2 |
| 4 | 1 |

Depicted are numbers of embryos
